# Oil in Water Microemulsions Loaded with Natural Products Curcumin and Mangiferin Are Effective Against *Fusarium verticillioides*

**DOI:** 10.3390/nano16090542

**Published:** 2026-04-29

**Authors:** Lucia Grifoni, Cristiana Sacco, Rosa Donato, Giulia Vanti, Maria Camilla Bergonzi, Anna Rita Bilia

**Affiliations:** 1 Department of Chemistry Ugo Schiff, University of Florence, Via Ugo Schiff 6, Sesto Fiorentino, 50019 Florence, Italy; lucia.grifoni@unifi.it (L.G.); giulia.vanti@unifi.it (G.V.); mc.bergonzi@unifi.it (M.C.B.); 2 Department of Health Sciences, University of Florence, Viale G.B. Morgagni, 50134 Florence, Italy; cristiana.sacco@unifi.it (C.S.); rosa.donato@unifi.it (R.D.)

**Keywords:** microemulsions, natural polyphenols, curcumin, mangiferin, phase diagram, dynamic light scattering, stability, *Fusarium verticillioides*, antifungal activity

## Abstract

The search for harmless alternative solutions to protect crops has become urgent and has recently attracted widespread attention from researchers around the world focusing on natural polyphenols, which represent a treasure chest of molecules with potent activities. Due to the low water solubility of polyphenols, microemulsions were selected as nanovectors. Curcumin and mangiferin solubility in different excipients was evaluated by HPLC. Microemulsion was developed using pseudo-ternary phase diagrams. Sizes and polydispersity of microemulsion globules were evaluated by dynamic light scattering. Activity against *Fusarium verticillioides* was evaluated by a microdilution method. Vitamin E acetate was selected as the oily phase, Transcutol P as cosurfactant and Tween 80 as surfactant. S_mix_ was composed of Transcutol P and Tween 80 in a 1:2 gravimetric ratio and combined with oil-phase vitamin E acetate at a weight ratio of 3:1. Microemulsions were loaded with 5 mg/mL of each polyphenol and recovery results were 99.5% and 99.3% for curcumin and mangiferin, respectively. Sizes of the lipid phase were 121.7 ± 29.2 nm and 172.6 ± 19.3 nm, respectively, for mangiferin and curcumin microemulsions. *F. verticillioides* was very susceptible to both microemulsions with a very high activity at a dose of 0.9 mg/mL (log-4 reduction), evidencing a possible use of these nanoformulations to protect crops from *F. verticillioides*.

## 1. Introduction

Synthetic pesticides are chemical substances employed to control or eliminate organisms that are harmful to plants, including insects, microorganisms, fungi, and weeds. Nowadays, they are extensively used in agriculture to protect and enhance crop productivity. Despite their widespread use, a substantial body of literature highlights the adverse effects of pesticides on human health and the environment. Undeniably, decades of pesticide application have led to their dissemination into the atmosphere and contamination of soil, water, and food sources, raising concerns regarding both acute and chronic toxicity in humans. The nature and severity of toxic effects are closely linked to the specific type of pesticide and the route of exposure. Documented toxicological outcomes include neurotoxicity, mutagenicity, carcinogenicity, teratogenicity, and endocrine disruption [1]. The significant increase in the use of chemical pesticides to combat crop diseases has created a growing awareness among many consumers because of the side effects of these pesticides on human health and the environment. Indeed, pesticide residues have become the focus of European bodies responsible for food safety [2].

The European Union has prioritized the mitigation of environmental and health risks associated with pesticide use, aiming to protect soil, water, air quality, and biodiversity. Through its vision for a resilient and competitive agri-food system, the EU has established stringent regulatory standards for plant protection products, which also apply to imported agricultural goods. In 2020, the European Commission proposed a 50% reduction in the use and risk of chemical pesticides by 2030; however, geopolitical challenges and strong opposition from agrochemical lobbying groups hindered its implementation. Consequently, the proposal was withdrawn in February 2024, raising uncertainty about the achievability of the EU’s pesticide reduction targets [3,4].

**Figure 1:**
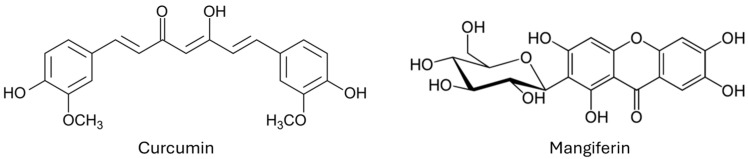
Chemical structures of curcumin and mangiferin.

Within this scenery, the search for alternative/integrative low-toxic molecules to protect crops has become urgent and has recently attracted widespread attention from researchers around the world focusing on natural products, which represent a treasure chest of molecules with potent activities. In this study we have selected several polyphenols, and from a preliminary screening, curcumin and mangiferin (Figure 1) were the most active against *Fusarium verticillioides*.

Curcumin (diferuloylmethane) is the main constituent (about 5% dry weight basis) of the rhizome of *Curcuma longa* L. (family Zingiberaceae), largely diffused as a food ingredient but it is also included in several pharmacopoeias. Other *Curcuma* species contain curcumin, and the percentage is comparatively similar or less [5]. Some emerging green technologies have been reported for curcumin extraction and isolation, as summarized in two very recent reviews [6,7]. The most interesting green technologies are represented by supercritical fluid extraction, ionic liquid-based extraction, and particularly natural deep eutectic solvents combined with ultrasonic probes, which are able to obtain very high extraction yields of more than 90% [6].

Mangiferin (6-C-glycosyl of 1,3,6,7-tetrahydroxyxanthone) represents the principal polyphenol of the peel, pulp, and seeds of mango, the fruit of *Mangifera indica* L. (family Anacardiaceae), but it is also present in the leaves [8]. A recent study has reported a new green and efficient extraction method of mangiferin, with hot water at 80 °C, from whole mango fruit (epicarp, endocarp, mesocarp, and seed). The mesocarp represented by the fleshy edible part of the fruit gave the highest amount of mangiferin (12.42 mg/g pulp) and the amount of extracted mangiferin per gram of fruit was 10.63 in total [9]. In addition, a study evidenced that the leaves can be extracted with a green process (water using a microwave, extraction time 5 min, plant bulk to solvent ratio 1:20 and microwave power of 272 W) obtaining superior amounts of mangiferin with a yield of 55 mg/g leaves [10].

Both curcumin and mangiferin are very safe molecules and can really represent valid alternatives to common synthetic pesticides. Indeed, curcumin has been shown to be safe at doses of up to 12 g/day in humans [5], while oral administration of mangiferin at doses ranging from 250 to 1000 mg/kg for 28 days did not cause any significant clinical or hematological changes in rats [11]. Previous studies evidenced that curcumin is effective against both Gram-negative and Gram-positive bacteria, various viruses, and numerous fungi [5,12,13,14]. Recently, curcumin was successfully tested against *Botrytis cinerea* spores [15] and curcumin-loaded nanoliposomes were tested against *Alternaria alstroemeriae* [16].

Additionally, it is reported that an ethanol extract of *Curcuma longa* possesses powerful antifungal activity, including several *Fusarium* species, namely *F. graminearum*, *F. chlamydosporum*, *F. tricinctum*, *F. culmorum*, and *F. oxysporum*, evidencing how curcumin is a main active constituent [17]. Similarly, a study reported the activity of a methanol extract of *Curcuma longa* against *Fusarium solani sensu lato*, evidencing the significant activity of curcumin as one of the main constituents [18]. Finally, in a more recent study focusing on the encapsulation of a derivative of curcumin, tetrahydrocurcumin, using a starch derivative and chitosan, the authors demonstrated promising antifungal properties against *Fusarium graminearum* [19]. According to Lee & Lee, the underlying mechanism of curcumin antifungal action is the disruption of the fungal plasma membrane inducing leakage of intracellular components through the flappy membrane [20].

Mangiferin has been also associated with antifungal properties [21,22] and it has been recently shown activity against postharvest fungal pathogens, including *Botrytis cinerea, Colletotrichum gloeosporoides*, and *Rhizopus stolonifer,* evidencing that mangiferin is a promising antifungal compound with potential applications in the food industries [23]. A dual mechanism for the antifungal activity was recently assessed [24]. Mangiferin acts simultaneously by disrupting both cell wall biosynthesis and vacuolar homeostasis, highlighting its potential as a multitarget antifungal agent.

Since curcumin and mangiferin, like other polyphenols, are poorly soluble in water, the development of suitable formulations, i.e., safe for users and the environment, effective at low dosages, stable during storage and use, easy to handle and apply, and designed to minimize off-target movement through specific physical and chemical properties, like controlled water solubility, is compulsory. In the present study, an oil in water microemulsion was selected as vector of curcumin and mangiferin because the superiority when compared with conventional formulations based on petroleum and organic solvents. Microemulsions have been studied extensively as vehicles for formulation of pesticides because of their unique advantages. They are thermodynamically stable and isotropic formulations, obtained spontaneously by mixing the excipients and active molecule with water. Nanodroplets are in the range of 30–100 nm, resulting in a large surface area, leading to better contact of the active ingredient with the fungi, and enhancing polyphenol delivery and reducing environmentally persistent organic solvents. Pesticide formulation based on microemulsions generally offers long-term thermodynamic stability, low viscosity, cost saving, and excellent attractiveness [25]. Selection of safe excipients was based on solubility of both curcumin and mangiferin and microemulsion was developed by titrating water into an oil–surfactant mixture with a pseudo-ternary phase diagram. After loading the formulations with curcumin and mangiferin, they were fully characterized and tested for their anti-*Fusarium* activity, making a comparison between unformulated polyphenols and the microemulsions loaded with mangiferin and curcumin.

## 2. Materials and Methods

### 2.1. Reagents and Solvents

Formic acid was from Sigma–Aldrich (Milan, Italy). Vitamin E acetate (alpha.-Tocopheryl acetate) was purchased from ACEF. Curcumin (purity 94%) was from Galeno (Prato, Italy). Mangiferin (purity > 98%), Tween 20, Tween 80, Tween 60 and HPLC grade acetonitrile were purchased from Merck (Rome, Italy); Transcutol P and Labrasol were gifts from Gattefossé (Saint Priest, France). All reagents were used as received, without further purification. Distilled water was obtained using a Milli-Q Advantage A10 system (Merck Millipore, Darmstadt, Germany). Amphotericin B was from Thermofisher Diagnostics SpA. Sabouraud Dextrose Broth (SDB) and Sabouraud Dextrose Agar (SDA) were from Oxoid, Thermo Scientific Diagnostics, Rodano, Milan, Italy.

### 2.2. HPLC-DAD Analysis to Evaluate Curcumin and Mangiferin Solubility in Different Vehicles, and Recovery and Stability of Developed Microemulsions

Curcumin and mangiferin were analyzed by HPLC-Photo Diode Array (PDA) Detector using a Waters Alliance 2695 Liquid Chromatograph (Waters Corporation, Milford, MA, USA) with Waters 2996 PDA Detector. Acquisition was from 245 to 600 n. The chromatographic profiles were registered at 427 nm for curcumin and at 319 nm for mangiferin. A Waters XTerra C18 column (150 × 3 mm, 5 μm; Waters Corporation, Milford, MA, USA) maintained at 25 °C was used for the analysis. Eluents were H_2_O (A, 0.1% formic acid) and acetonitrile (B) at a flow rate of 0.45 mL/min. The mobile phase composition is reported in Table 1. Method validation was according to international regulatory guidelines [26,27]. Retention time of mangiferin was 2.37 min, and retention time of curcumin was 10.58 min.

### 2.3. Solubility Studies of Curcumin and Mangiferin in Different Surfactants

To develop microemulsions, the solubility of curcumin and mangiferin were determined in different surfactants and co-surfactants. An excess amount of curcumin or mangiferin (100 mg) was added to 2 mL of surfactant. Each mixture was shaken at 25 ± 2 °C for 24 h, and then was centrifuged at 13,148× *g* for 10 min. In the supernatant phase curcumin or mangiferin concentration was quantified by HPLC-DAD, after 10-fold dilution with methanol/dichloromethane (6:4). The analyses were performed in triplicate.

### 2.4. Development of Microemulsion

Tween 80 and Transcutol P were selected as surfactant and co-surfactant, respectively, based on solubility studies. Various S_mix_ blends were tested by combining gravimetric ratios of 1:1, 1:2, and 1:3 of Transcutol P and Tween 80. These S_mix_ blends were then combined with oil-phase vitamin E acetate at various weight ratios (1:9, 2:8, 3:7, 4:6, 5:5, 6:4, 7:3, 8:2 and 9:1). Each mixture was stirred magnetically at 50 ± 2 °C for five minutes, with the temperature being monitored via a temperature sensor. Each mixture was then titrated dropwise with ultrapure water. After the initial titration, the resulting lipophilic mixtures were cooled to 35 ± 2 °C and further titrated with water dropwise while being stirred at 500 rpm using a magnetic stirrer set to maintain a constant temperature of 35 ± 2 °C.

The microemulsion region was identified by constructing pseudo-ternary phase diagrams using OriginPro software. During water titration, changes in sample appearance were monitored visually to classify the resulting systems as transparent microemulsions, emulsions, gels or turbid mixtures. The microemulsions were then allowed to stabilize at 35 ± 2 °C for 10 min before being left to cool to room temperature (21 ± 2 °C) under gentle stirring [28].

### 2.5. UV/Visible Spectrophotometric Percentage Transmittance Characterization

Transparency of micro emulsion formulation was determined by measuring percentage transmittance through UV/Visible spectrophotometer (DAD 8453 Agilent Technologies) at 650 nm with distilled water taken as blank. Three replicate assays were performed for each sample (3 mL) as previously reported [29].

### 2.6. Physical Characterization of the Microemulsions

Microemulsions were evaluated for their globule size and polydispersity index using Dynamic Light Scattering (DLS) [30]. All measurements were performed using a Nano ZS Zetasizer (Malvern Instruments Ltd., Malvern, UK) equipped with a He-Ne laser of 532 nm at a scattering angle θ of 173°. Quartz standard cuvettes were used for size measurements. Experiments were performed at 25 °C and samples were diluted 10-fold in ultrapure water prior to measurements. The analyses enable the determination of the samples’ average hydrodynamic diameter (Size, nm) and polydispersity index (PDI, a dimensionless parameter). All data were processed using the Cumulants method defined in the International Standards and recognized by Health Agencies (ISO 22412:2025; ASTM E3247-20). Each measurement was performed using square polystyrene cuvettes for DLS.

### 2.7. Scanning Transmission Electron Microscope (STEM) Analysis

STEM analysis was performed as previously reported [31] Briefly, microemulsions and the corresponding diluted dispersion (obtained by 20-fold dilution in ultrapure water) were analyzed by the Scanning Electron Microscope (SEM) Gaia 3 (Tescan s.r.o, Brno, Czech Republic), FIB-SEM (Focused Ion Beam-Scanning Electron Microscope), operating in high-vacuum mode with electron beam voltage of 20 kV and Bright-field Transmission Electron Microscope (TEM) detector. Gaia 3 was equipped with an EDS-X-ray microanalysis system (EDAX AMETEK GmbH, Weiterstadt, Germany) TEAM EDS Basic Software Suite TEAM™ and was delivered with a STEM (Scanning Transmission Electron Microscopy) detector, which provides a complementary method for image acquisition of transmitted electrons. The detector consists of several semi-conductor sensors for bright-field and dark-field imaging. By placing the detection system below the specimen, the transmitted electron signal can be collected.

### 2.8. Solubilization of Curcumin and Mangiferin into Microemulsions and Characterization

Curcumin- and mangiferin-loaded microemulsions were prepared by dissolving the polyphenols into the oil-S_mix_ mixture, adding the required quantity of water, and stirring to form a clear and transparent dispersion. The resulting microemulsions were tightly sealed and stored at +4 °C temperature. Stabilization and cooling procedures of the resulting microemulsions were done according to Section 2.4. Globule size and polydispersity index were evaluated by DLS as reported in Section 2.6.

The amount of curcumin and mangiferin effectively loaded into the microemulsion and recovered at the end of the preparation procedure is referred to as the recovery. It is evaluated by dissolving the microemulsion globule by 100-fold dilution with methanol. The obtained samples were vortexed and immersed in the ultrasonication bath for 5 min at 25 ± 2 °C to improve the globule dissolution and polyphenol extraction in the organic solvent. The samples were centrifuged for 5 min at 14,000 rpm to separate any undissolved microemulsion components and were analyzed by HPLC-PDA, as described in Section 2.2. Six different samples, using a triplicate each time, were evaluated for the recovery [29]. Recovery percentage (R%) is expressed as the percentage of the recovered amount compared with the initial weighted amount of the drug ring according to Equation (1):

(1) $$Recovery\;\%\;=\;\frac{detected\;mg}{total\;mg}\;*\;100$$

### 2.9. Evaluation of Stability of MA-ME and CU-ME

Centrifugation was performed at 12,830× *g* for 30 min at 25 °C to assess the physical stability of MA-ME and CU-ME. Preliminary chemical and physical stability of MA-ME and CU-ME, stored at 21 ± 2 °C away from the light, was monitored for 20 days. Physical stability was also assessed by UV/Visible spectrophotometric percentage according to the methods reported in Section 2.5, whereas mangiferin and curcumin concentration, expressed as recovery percentage, were determined by HPLC-PDA as described in Section 2.2.

### 2.10. Antifungal Assay

The antifungal activity was evaluated using a microdilution method against a *F. verticillioides* using a strain isolated from Amaranth flour. Its taxonomical classification was confirmed by sequencing EF1 and ITS-LR. The analysis was performed via the Basic Local Alignment Search Tool (BLAST) to find regions of local similarity between sequences, and the results showed 100% similarity for all the genes belonging to *F. verticillioides* [32]. The *Fusarium* strain used in the tests was kept in water at 4 °C in the fridge. Routine steps were taken. The strain was grown in Sabouraud Dextrose Broth (SDB) and incubated for 5 days at 28 °C. After incubation, the revived strain was grown on Sabouraud Dextrose Agar (SDA) at 28 °C for 5 days. The cells were harvested by adding 10 mL of sterile distilled water containing 0.05% Tween 80 and scraping the surface of the culture. The fungal concentration was evaluated both by spectrophotometric reading (BioPhotometer Eppendorf srl, Zevenhuizen, The Netherlands) (OD 600) and by counting subcultures on SDA incubated at 28 °C for 5 days. The concentration of the fungal stock solutions was between 1.9 and 4.2 × 10^6^ colony-forming units (CFU/mL). In this study, the 20 μL inoculum of fungal strain used in the microtiter plate had a concentration between 3.8 and 8.4 × 10^4^. Scaling amounts of curcumin, mangiferin, unloaded microemulsions (blank or empty microemulsions, composed of 10% *v*/*v* vitamin E acetate, 10% *v*/*v* Transcutol P, 20% *v*/*v* Tween 80 and 60% *v*/*v* of water), mangiferin-loaded and curcumin-loaded microemulsions were used. Scalar concentrations of CU-ME and MA-ME were added to the wells to obtain a percentage concentration between 90% and 50% in the SDB culture medium. A total of 20 μL of the fungal stock solution was added to all wells. Negative and positive controls were prepared. The antibiotic amphotericin B was included as a positive control, while SDB was the negative control. The empty microemulsion was also tested on the strain to exclude its possible antifungal activity, as it contains surfactants. The plate was incubated for 24 h at 28 °C. Subsequently, the entire content of the wells was included in Petri dishes using SDA and incubated at 28 °C for 5 days. The test was repeated twice in triplicate. The ability to reduce the number of *F. verticillioides* was calculated by logarithmic reduction, i.e., a reduction of 4 logarithms corresponded to a reduction of 99.99% and 5-log reduction corresponded to 99.999% reduction. A product able to achieve a reduction of at least 4 logarithms can pass the EN 1275 standard [33,34]. The calculation of the logarithmic reduction can be obtained from the following formula:

(2) $$Logarithmic\;reduction\;=\;\log_{10}\left(A\right)\;-\;\log_{10}\left(B\right)$$

where *A* is the number of viable microorganisms before treatment and *B* is the number of viable microorganisms after treatment.

### 2.11. Statistical Analysis

Results are reported as mean values ± SEM. Statistical significance of antifungal tests was assessed by Two-way ANOVA followed by Bonferroni’s post hoc test. The two factors were the sample treatment and concentrations. The first significance threshold for all tests was set for *p* < 0.05 (*), and the second threshold was set for *p* < 0.01 (**).

## 3. Results

### 3.1. HPLC-DAD Method

Before formulating curcumin and mangiferin, a suitable HPLC-DAD analytical method for their quantification was developed and validated. No interferences with the excipients and final formulation were observed at the selected detection wavelengths (427 and 319 nm). Good linearity with a correlation coefficient (*R*^2^) of 0.9998 in the range of 0.1–100 μg/mL of both constituents was found. The accuracy of the method was evaluated by adding the standard solution of 0.05, 0.1, 1, and 10 (μg/mL) to known sample solutions. In robustness, the results remain unaffected by small variations in the analytical parameters, which shows the robustness of the method. Limit of detection (LOD) and limit of quantification (LOQ) were calculated by determination of the signal-to-noise ratio, in accordance with the ICH guidelines (EMA, 1995). The signal-to-noise ratio was 3 for estimating the LOD, while the signal-to-noise ratio was 10 for estimating the LOQ. The limit of detection (LOD) and limit of quantification (LOQ) for curcumin were 0.010 μg/mL and 0.060 μg/mL, respectively. LOD and LOQ for mangiferin were 0.019 μg/mL and 0.085 μg/mL, respectively.

### 3.2. Selection of Vehicles

Vitamin E acetate was selected as the oily phase because vitamin E and its derivatives are widely used as inert ingredients in pesticide formulations acting as stabilizers and preventing oxidation. Vitamin E derivatives can protect biological membranes from damage caused by pesticides and oxidative stress, mitigating some of the toxic effects of the pesticide. Due to its safety profile when used as an inert ingredient, the U.S. Environmental Protection Agency has exempted vitamin E derivatives from the requirement of a tolerance for use in pesticide formulations. Remarkably, vitamin E acetate is more stable to light and air than vitamin E itself, making it a preferred choice for formulations [35]. Solubility of curcumin and mangiferin in the different vehicles was assessed by HPLC-PDA analyses to select those with the highest solubility. Accordingly, Transcutol P was selected as the co-surfactant because of the elevated solubility of both curcumin and mangiferin, allowing for a high polyphenol concentration within the microemulsion (Table 2). Noticeably, Transcutol P helps stabilize the microemulsion by extending the microemulsion domain area, which is the range of component concentrations where a stable, single-phase microemulsion can be formed by improving the flexibility of the oil–water interface. Additionally, it enhances oil penetration and can also act as a penetration enhancer. It can better penetrate the surface of plant’s cuticle and cell walls, as well as fungal cells, more easily, increasing the overall efficacy of the treatment [36,37]. Several hydrophilic surfactants to formulate the microemulsion were evaluated for their solubilization of curcumin and mangiferin. Results are reported in Table 2 as the mean value of three replicates. Agreeing with the reported results, Tween 80 was the hydrophilic surfactant displaying the highest solubility of both curcumin (30 mg/mL) and mangiferin (36 mg/mL), and for this reason it was selected to developed both curcumin and mangiferin microemulsions.

### 3.3. Microemulsion Development

**Figure 2:**
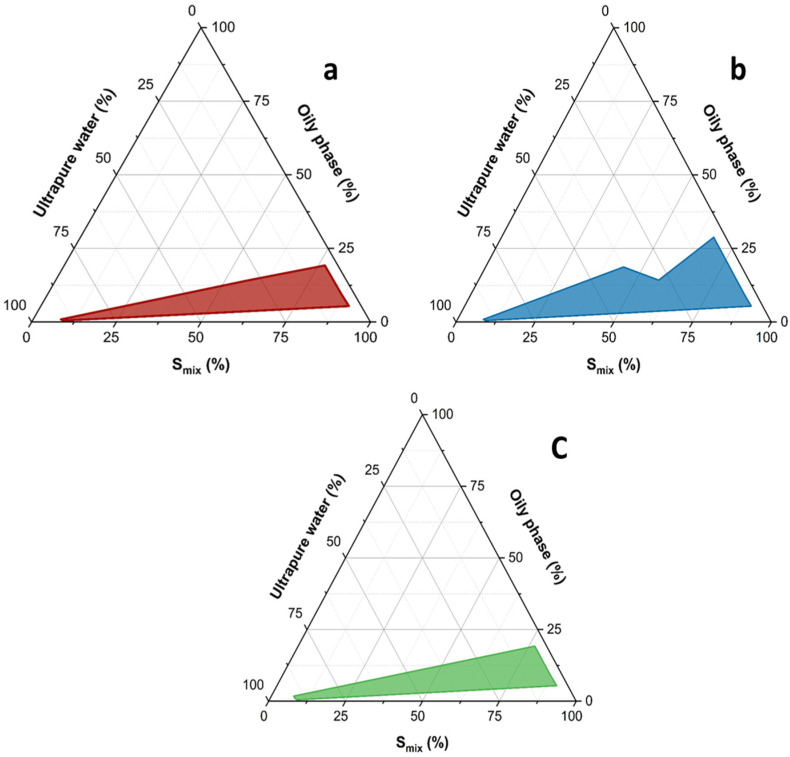
Pseudo-ternary phase diagrams of the microemulsions developed using the three surfactant blends (S_mix_), namely Transcutol P and Tween 80 with 1:1 (**a**), 1:2 (**b**) and 1:3 (**c**) ratios. Colored areas represent stable, one-phase microemulsion regions.

Among the different nanoencapsulation approaches to formulate pesticides, microemulsions and nanoemulsions are the most promising self-emulsifying colloidal systems as they are economical, easiest to formulate and handle. Microemulsions form spontaneously without the use of a high-pressure homogenizer or high shear instrument [38]. In the case of nanoemulsions, which are not thermodynamically susceptible to Ostwald ripening, they are not susceptible to creaming, flocculation, and other physical instability problems associated with emulsions [23]. Three microemulsions made of vitamin E acetate, Transcutol P and Tween 80 were developed by a pseudo-ternary diagram, using OriginPro software (Figure 2a–c).

To understand the phase behavior and the transition boundaries of the present multicomponent ME system, the pseudo-ternary phase diagrams were drawn using three surfactant blends. The blend (S_mix_) of Transcutol P and Tween 80 in a 1:1, 1:2 and 1:3 gravimetric ratios (Figure 2a–c), obtained from three pseudo-ternary phase diagrams, had the following weight ratios of oil/S_mix_: 0:100, 5:95, 10:90, 20:80, 30:70, 40:60, 50:50, 60:40, 70:30, 80:20 and 90:10, as elaborated by testing. Pseudo-ternary phase diagrams were constructed using a water titration method to obtain the concentration range of all components that can form microemulsions. Each oil-S_mix_ mixture was diluted under stirring dropwise at 35 °C with water. After equilibrium, each sample was visually checked, monitoring appearance to identify the resulting systems as transparent microemulsions, emulsions, gels or turbid mixtures. The experiment results showed that the regions of MEs in the pseudo-ternary phase diagram using different S_mix_ were different and the final selection of microemulsion was based on the surface of the microemulsion domain area that was more extended. From the evaluation of the colored regions of Figure 2a–c, the blend using as S_mix_ with a 1:2 gravimetric ratio of Transcutol P and Tween 80 resulted in the most efficiency in giving the greatest microemulsion domain area. Finally, the selected microemulsion for further studies was that obtained with the ratio 1:3 of vitamin E acetate and S_mix_ (1:2). The composition of the carefully chosen microemulsion for loading both curcumin and mangiferin was the following: 10% *v*/*v* vitamin E acetate, 10% *v*/*v* Transcutol P, 20% *v*/*v* Tween 80 and 60% *v*/*v* of water.

### 3.4. Development of Curcumin- and Mangiferin-Loaded Microemulsions

Mangiferin- and curcumin-loaded microemulsion were obtained by mixing these polyphenols to the oily phase containing surfactants before water titration, then adding water incrementally to this mixture. Mangiferin- and curcumin-loaded microemulsions were obtained when the system became clear and stable indicating the formation of the microemulsion. Compositions of the microemulsions loaded with polyphenols are reported in Table 3. The theoretical content of polyphenols in the final formulation was 5 mg/mL of microemulsion. The recovery percentage of curcumin and mangiferin was assessed by HPLC-PDA as reported in the experimental part. Curcumin recovery results were 99.13 ± 3.20%, while mangiferin recovery was 98.23 ± 2.61%.

To assess the optimized microemulsion formulation reproducibility, three independent batches of CU-ME and MA-ME were prepared using the same procedure. Firstly, the samples were analyzed by visual inspection for transparency, clarity, and lack of phase separation. Additionally, percentage transmittance of CU-ME and MA-ME was measured using the UV/Vis spectrophotometer at 650 nm, a region without absorbance for both curcumin (λ*_max_* at 427 nm) and mangiferin (λ*_max_* at 319 nm, other peaks were at 257 and 363 nm). All the samples had high values (near 100%) for percentage transmittance, a key physicochemical parameter. The CU-ME percentage transmittance value was 99.6 ± 0.09, and the MA-ME percentage transmittance value was 99.3 ± 0.11, demonstrating high ME reproducibility.

### 3.5. Physical Characterization of CU-ME and MA-ME

The hydrodynamic diameter and polydispersity of the globules of the developed microemulsions were determined by DLS as reported in the experimental part and reported in Table 4. The dimensions of the microemulsion globules loaded with mangiferin were lower than those obtained with the microemulsion loaded with curcumin (about 122 nm versus 173 nm). In these oil-in-water (O/W) microemulsions the polyphenols’ presence at the oil–water interface can affect the interfacial tension and the packing of surfactants around the droplet. This can influence the stability of the droplet and its size. If the compound possesses amphiphilic properties, it can act as a secondary surface-active agent or co-surfactant. Truly, mangiferin is a C-glycosyl compound, and the sugar represents the hydrophilic portion, while the xanthone core is lipophilic, imparting light amphiphilic properties that can justify in part the differences in globule size of the two microemulsions. Additionally, the small sizes of the globules improve the curcumin and mangiferin wetting, spreading, and permeability on leaf surfaces, increasing their effectiveness [23,36].

The polydispersity index values obtained as a mean of three replicates were less than 0.3 for both microemulsions, indicating a very stable and uniform system of similar-sized droplets [27].

**Figure 3:**
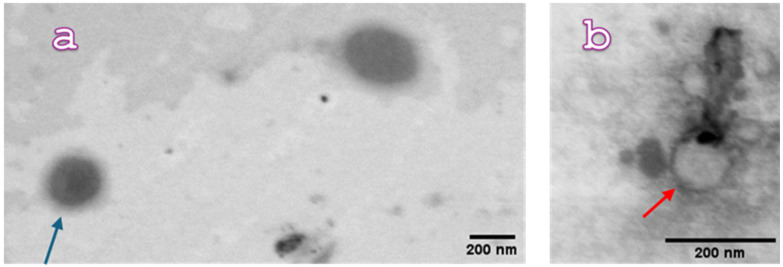
Pictures of: (**a**) CU-ME (curcumin-loaded ME, arrow light blue), bar = 200 nm; (**b**) MA-ME (mangiferin-loaded ME, arrow red), bar = 200 nm, obtained by STEM analysis.

CU-ME and MA-ME were morphologically analyzed by the Scanning Electron Microscope (SEM) Gaia 3 with a bright-field TEM detector. (Figure 3) Microscopy observation of the formulations confirmed the DLS results. The picture of CU-ME (Figure 3a) clearly shows droplets with a spherical shape and dimensions comparable to those obtained by DLS (ca. 170 nm, arrow light blue). Micrographs of MA-ME (Figure 3b) also displayed spherical droplets with similar dimensions of those obtained by DLS (ca. 120 nm, arrow red).

### 3.6. Stability of CU-ME and MA-ME

To confirm CU-ME and MA-ME stability through accelerated testing, centrifugation was carried out as reported in the experimental part. No phase separation was found for all the samples, which retained high homogeneity, demonstrating that optimized CU-ME and MA-ME are physically stable. Furthermore, to assess the presence of eventual phase separation, both MEs were investigated for 20 days after storage at 21 ± 2 °C by transmittance percentage (% T) using a UV-Vis spectrophotometer as reported in in the experimental part. The results are reported in Table 5. The UV-Vis analyses showed high % T values during the 20 days of testing, indicating the transparency/clarity of both MEs and evidencing that optimized CU-ME and MA-ME were physically stable, retaining homogeneity without phase separation. Furthermore, MEs were also evaluated by HPLC-DAD, as reported in the experimental part, to evaluate the percentage residual content (% C) of curcumin and mangiferin in the tested CU-ME and MA-ME. The results reported in Table 5 provide evidence for the great chemical stability of both CU-ME and MA-ME.

### 3.7. Antifungal Activity

The antifungal tests were carried out using amphotericin B as the positive control and SDB as the negative control. In our study 20 μL inoculum of the fungal strain was used in the microtiter plate, having a concentration between of 8.8 × 10^4^ and 2.4 × 10^5^ CFU/20 µL. Samples were tested against *F. verticillioides* using a microplate dilution method. Antifungal activity of curcumin, mangiferin, the unloaded microemulsions, MA-ME and CU-ME, negative and positive controls was tested. The investigation of the antifungal properties of the developed MEs was the first step of the study to evaluate their possible contribution to the antifungal activity. The empty ME (namely 10% *v*/*v* vitamin E acetate, 10% *v*/*v* Transcutol P, 20% *v*/*v* Tween 80 and 60% *v*/*v* of water) was diluted with SBD obtaining mixtures containing from 5 to 60%, namely 5, 10, 20, 30, 40, 50 and 60%, and tested using 8.8 × 10^4^ CFU/20 µL. None of the investigated samples displayed antifungal activity against *F. verticillioides*, suggesting that vitamin E acetate and the two surfactants present in the formulation have no direct activity against the fungi by interacting with cytoplasmic membrane or other mechanisms, which can result in the fungicidal properties. According to our results, great biocompatibility of the empty nanoformulation was found, providing evidence for the noticeable safety of these nano-drug delivery systems. The study continued with the evaluation of the antifungal activity of pure curcumin and mangiferin. Briefly, mother solutions of curcumin and mangiferin were prepared using DMSO. Successively, different concentrations of both polyphenols obtained by scalar dilutions of mother solutions with SBD were evaluated against *F. verticillioides* using 2.4 × 10^5^ CFU/20 µL, as reported in Table 6. The results are the average of three tests repeated in triplicate.

Mangiferin and curcumin displayed a similar antifungal potency, evidenced by a log 5 reduction up to a minimal concentration of about 14.5 μg/µL. At lower concentrations, namely around 12.65 μg/µL, a log 4 reduction is obtained (Table 6).

The antifungal activity of the microemulsions loaded with mangiferin (MA-ME) and curcumin (CU-ME) was also tested using scalar amounts of microemulsions, with concentrations of 4.50, 4.00, 3.50, and 3.00 μg/μL (Table 7). Results are reported as the average of three tests repeated in triplicate and demonstrated a 4-log reduction in fungal colony-forming units (CFUs) at a dose of 4.50 μg/μL for both formulations. At lower doses (4.00 and 3.50 μg/μL), antifungal activity was still observed, corresponding to a 3-log reduction in the *Fusarium verticillioides* strain. A 3-log reduction should still be regarded as a positive outcome in terms of antifungal activity, considering that the initial concentration of the strain was nearly 5 log (8.8 × 10^4^). These findings were very interesting, demonstrating how the nanoformulations are more active than the solutions of the single polyphenols. Indeed, a logarithmic reduction of *F. verticillioides* of 5 for each polyphenol formulated in the microemulsion was reached at a dose of about 320 times less than the solution of the pure polyphenols. The enhanced activity can be explained because the microemulsion’s tiny, stable droplets, which increase the surface area of the fungi for absorption, improving the penetration through the fungi cell membranes, as well as the presence of Transcutol P, which is well known as an enhancer penetration substance in biological membranes, enhancing both the solubilization and absorption rate of actives. These results highlight the potential of MA-ME- and CU-ME-based nanosystems as effective antifungal agents that are more efficient compared to traditional pesticides.

**Figure 4:**
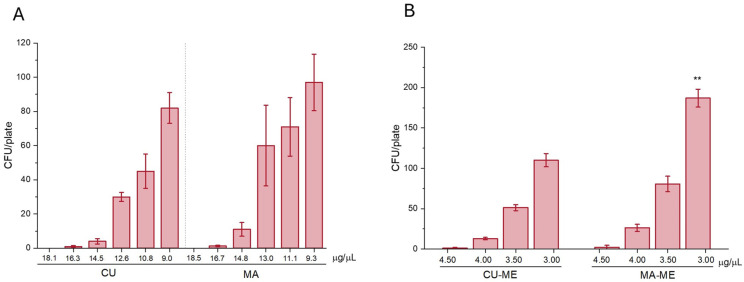
*F. verticillioides* antifungal activity expressed as CFU/plate of tested samples. (**A**) Effect of curcumin (CU) and mangiferin (MA) at different tested concentrations. (**B**) Effect of microemulsion formulations containing curcumin (CU-ME) and mangiferin (MA-ME). Data are expressed as mean ± SD (*n* = 5). Statistical significance between CU-ME and MA-ME groups was evaluated using a pair sample t-test, and significant differences are indicated by asterisks (**, *p* < 0.01).

Finally, in Figure 4A,B, the *F. verticillioides* antifungal activity is reported as the residual CFU/plate of the different sample concentrations. The overall results evidenced that fungal growth progressively increases as the concentration of curcumin and mangiferin decrease, indicating a reduction in *F. verticillioides* antifungal activity. Unformulated curcumin (CU) and mangiferin (MA) dispersion showed a similar trend without significant differences. A direct comparison between the pure compounds and the ME formulations was not possible because of the different tested concentrations. Concerning the antifungal activity of ME formulations, the comparison between CU-ME and MA-ME shows that only MA-ME exhibits a significantly (** *p* < 0.01) lower antifungal effect at 3.00 μg/μL when compared with the same concentration of CU-ME.

## 4. Conclusions

In this study, O/W MEs based on curcumin and mangiferin were developed as an innovative formulation to fight *F. verticillioides*. The proposed MEs are formulated using food-grade ingredients with a high content of water, minimum surfactant and oil concentrations, and encapsulating 5 mg/mL curcumin and mangiferin, which are safe polyphenols. The high efficacy and multitarget action of curcumin may reduce the likelihood of resistance development in synthetic pesticides by acting as single mechanism.

ME-based pesticides are considered better than nanoemulsion-based pesticides as they are thermodynamically stable. The developed CU-ME and MA-ME are easy to scale up, completely biodegradable, and less expensive than formulations obtained with organic solvents. In addition, O/W MEs are not flammable and have a low viscosity, making them safe and easy to transport, store, and handle. The oily internal phase has antioxidant properties due to the presence of vitamin E acetate, contributing to the stability of the formulation, and preserving the biological properties of curcumin and mangiferin.

Collectively, our in vitro findings reveal the effective concentrations, supporting further development involving a multi-stage approach against *Fusarium* infections under field and storage conditions. After the present lab tests, future studies should be moved to controlled greenhouse/pot trials (soil/seed treatments, monitoring disease index) for efficacy, field trials (seed treatment, foliar spray) and finally post-harvest storage tests for protection against *Fusarium* to assess real-world performance against this fungus.

## Figures and Tables

**Table 1:** Gradient system used for the HPLC-PDA analyses. Eluent A was H_2_O (0.1% formic acid), and eluent B was acetonitrile.

Time	Eluent A	Eluent B
0.00	75.0	25.0
10.00	25.0	75.0
15.00	25.0	75.0
16.00	75.0	25.0
26.00	75.0	25.0

**Table 2:** Solubility (mg/mL) of curcumin and mangiferin in different vehicles calculated by HPLC-PDA. For each surfactant the HLB is reported in parenthesis.

Vehicles (HLB)	Curcumin Solubility	Mangiferin Solubility
Tween 20 (16.7)	8 mg/mL	26 mg/mL
Tween 80 (15.0)	30 mg/mL	36 mg/mL
Tween 60 (14.9)	13 mg/mL	31 mg/mL
Transcutol P	95 mg/mL	29 mg/mL
Labrasol (12)	15 mg/mL	23 mg/mL
Water	0.1 mg/mL	0.8 mg/mL

**Table 3:** Compositions of the developed microemulsions (MEs) selected for antifungal evaluation.

ME	Vitamin E Acetate (% *v*/*v*)	Transcutol P (% *v*/*v*)	Tween 80 (% *v*/*v*)	Water (% *v*/*v*)	Curcumin (% *w*/*v*)	Mangiferin (% *w*/*v*)
CU-ME	10	10	20	60.0	0.5	
MA-ME	10	10	20	60.0		0.5

ME: microemulsion; CU-ME: curcumin-loaded microemulsion; MA-ME; mangiferin-loaded microemulsion.

**Table 4:** Size and polydispersity of the oily phase of the developed microemulsions (MEs). Data are expressed as mean ± SD (*n* = 3).

	Empty-ME	MA-ME	CU-ME
Size (nm)	95.2 ± 10.3 nm	121.7 ± 29.2 nm	172.6 ± 19.3 nm
Polydispersity Index	0.100 ± 0.009	0.280 ± 0.010	0.299 ± 0.009

MA-ME: Mangiferin microemulsion; CU-ME: Curcumin microemulsion.

**Table 5:** Transmittance percentage (% T) and percentage residual content (% C) of CU-ME and MA-ME during storage at 21 ± 2 °C. Data are expressed as mean ± SD (*n* = 3).

Day	CU-ME (% T)	CU-ME (% C)	MA-ME (%T)	MA-ME (% C)
0	99.91 ± 1.02	100.00 ± 0.00	99.81 ± 1.04	100.00 ± 0.01
10	100.12 ± 0.08	99.91 ± 0.03	99.91 ± 0.09	99.93 ± 0.08
15	99.64 ± 1.35	99.91 ± 0.06	99.72 ± 1.14	99.92 ± 0.12
20	99.53 ± 1.09	99.92 ± 0.01	99.82 ± 1.23	99.91 ± 0.23

MA-ME: Mangiferin microemulsion; CU-ME: Curcumin microemulsion.

**Table 6:** Logarithmic reduction in *F. verticillioides* CFU by curcumin and mangiferin.

*F. verticillioides*	Tested Curcumin Concentration (μg/μL)					
2.4 × 10^5^ (CFU/20 μL)	18.10	16.30	14.50	12.65	10.85	9.05
Log reduction	5	5	5	4	3	3
***F. verticillioides***	**Tested Mangiferin Concentration (μg/µL)**					
2.4 × 10^5^ (CFU/20 μL)	18.55	16.70	14.85	13.00	11.15	9.30
Log reduction	5	5	5	4	3	3

**Table 7:** Logarithmic reduction of *F. verticillioides* CFU by curcumin-loaded microemulsion (CU-ME) and mangiferin-loaded microemulsion (MA-ME).

*F. verticillioides*	Tested Curcumin Concentration in CU-ME (μg/μL)			
8.8 × 10^4^ (CFU/20 µL)	4.50	4.00	3.50	3.00
Log reduction	4	3	3	2
***F. verticillioides***	**Tested Mangiferin Concentration in MA-ME** (**μg/μL)**			
8.8 × 10^4^ (CFU/20 µL)	4.50	4.00	3.50	3.00
Log reduction	4	3	3	2

## Data Availability

The original contributions presented in this study are included in the article. Further inquiries can be directed to the corresponding author.
